# Real-world data on long-term outcomes in patients with T-cell lymphomas: a nationwide study of Korea

**DOI:** 10.1007/s44313-025-00095-1

**Published:** 2025-08-21

**Authors:** Dong Won Baek, Jung Min Lee, Youngeun Jang, Yunji Lee, Hee Jeong Cho, Joon Ho Moon, Hasung Kim, Hoseob Kim, Sang Kyun Sohn

**Affiliations:** 1 Department of Hematology/Oncology, Kyungpook National University Hospital, School of Medicine, Kyungpook National University, Daegu, Korea; 2 Department of Data Science, Hanmi Pharm. Co., Ltd, Seoul, Korea

**Keywords:** Lymphoma, T-cell, Chemotherapy, Stem cell transplantation, Survival

## Abstract

**Background:**

Peripheral T-cell lymphomas (PTCLs) are a rare and heterogeneous group of aggressive malignancies. This study aimed to comprehensively analyze patients diagnosed with PTCLs in Korea, evaluating treatment outcomes, including transplantation and long-term survival.

**Patients and methods:**

In this retrospective study, clinical data from the National Health Insurance Service on patients with PTCL were investigated. Most patients diagnosed with mature T-cell lymphomas and natural killer (NK)/T-cell lymphomas between January 2005 and December 2022 in Korea were included. Incidence rates of each subtype and survival outcomes of both treated and untreated patients were analyzed.

**Results:**

A total of 12,573 patients were analyzed. PTCL not otherwise specified (PTCL-NOS) and extranodal NK/T-cell lymphoma were the most frequently diagnosed, followed by angioimmunoblastic T-cell lymphoma (AITL). Compared to the general population, the relative survival rate was highest in anaplastic lymphoma kinase (ALK)-positive anaplastic large cell lymphoma. With a median follow-up of 6.7 years, the 3-year and 5-year progression-free survival (PFS) rates among treated patients were 44.0% and 39.5%, while the overall survival (OS) rates were 48.6% and 43.5%, respectively. Kaplan–Meier survival curves indicated that patients who added etoposide to the CHOP (cyclophosphamide, doxorubicin, vincristine, and prednisone) regimen showed improved PFS and OS. In addition, autologous stem cell transplantation significantly improved PFS and OS, particularly in the PTCL-NOS and AITL subtypes.

**Conclusion:**

Patients who received etoposide-containing CHOP-based regimens had improved treatment outcomes. The survival benefits of consolidative autologous stem cell transplantation (auto-SCT) were evident in PTCL-NOS and AITL.

**Supplementary Information:**

The online version contains supplementary material available at 10.1007/s44313-025-00095-1.

## Introduction

Peripheral T-cell lymphomas (PTCLs) comprise a heterogeneous group of mature T-cell and natural killer (NK)-cell neoplasms, generally presenting with aggressive clinical features. PTCLs are less common than B-cell lymphomas, accounting for 10–15% of all non-Hodgkin lymphomas [1, 2]. According to the 2016 World Health Organization (WHO) classification, nodal T-cell lymphomas include anaplastic lymphoma kinase (ALK)-positive and ALK-negative anaplastic large cell lymphoma (ALCL), angioimmunoblastic T-cell lymphoma (AITL), and PTCL not otherwise specified (PTCL-NOS). Among extranodal (EN) lymphomas, NK/T-cell lymphoma, nasal type, is a representative subtype [3]. In the 5th edition of the WHO classification, PTCLs have been further classified based on cell of origin, differentiation state, and other factors [4].

Treatment strategies for PTCL have remained largely unchanged for decades, with prognosis remaining poor. The 5-year survival rate is approximately 30–40% [5–8]. Several treatment-related issues remain unresolved, including the optimal first-line therapy—whether CHOP (cyclophosphamide, doxorubicin, vincristine, and prednisone) or CHOEP (CHOP plus etoposide)—the role of brentuximab vedotin (BV), and the benefits of up-front stem cell transplantation (SCT). Data from the Swedish Lymphoma Registry show that adding etoposide to CHOP improves progression-free survival (PFS) in patients under 60 years of age; however, the study, which included only 252 patients, reported no significant improvement in overall survival (OS) [5]. The ECHELON-2 trial demonstrated superior outcomes with BV plus CHP (cyclophosphamide, doxorubicin, and prednisone) over CHOP in patients with CD30-positive systemic ALCL [9, 10]. Standard chemotherapy followed by high-dose therapy and autologous SCT (auto-SCT) remains the preferred option for transplant-eligible patients with PTCL [11, 12]. However, a recent randomized phase 3 trial reported no survival advantage of up-front consolidative allogeneic SCT (allo-SCT) over auto-SCT [13].

A prior study reported long-term survival outcomes in patients with PTCL who underwent auto-SCT or allo-SCT using data from the Korean Society of Blood and Marrow Transplantation registry. Consolidative up-front auto-SCT demonstrated a survival benefit and was also effective in salvage settings. Allo-SCT remains a viable option for patients with relapsed or refractory disease. However, that study was limited to transplant recipients and did not include all PTCL cases in Korea [14]. Therefore, the present study aims to investigate the majority of patients diagnosed with PTCLs in Korea over approximately 20 years, using clinical data from the National Health Insurance Service (NHIS). We summarized the distribution of diagnoses, incidence, and treatment outcomes, including transplantation.

## Materials and methods

### Patients

In this retrospective study, clinical data of all patients with T-cell lymphoma from the NHIS were included. Regardless of age, most patients diagnosed with mature T-cell lymphomas and NK/T-cell lymphomas between January 2005 and December 2022 in Korea were selected by searching Korean Classification of Diseases (KCD) codes in the NHIS patient database. Diagnoses were classified based on the 2016 WHO classification [3]. Pathology slide review was not performed. Chemotherapy regimens and treatment schedules, including those involving transplantation, were determined by local physicians at each center. This study was approved by the Institutional Review Board of Kyungpook National University Chilgok Hospital (KNUCH 2023-01-004) and conducted in accordance with the Declaration of Helsinki.

### Statistical analyses

The incidence rate of each subtype and survival outcomes of both treated and untreated patients were analyzed. Regarding treatment outcomes, we mainly analyzed patients who received CVP, CHOP, CHOEP, EPOCH, VIPD (etoposide, ifosfamide, cisplatin, and dexamethasone), SMILE (methotrexate, ifosfamide, l-asparaginase, etoposide, and dexamethasone) regimens, and CCRT (concurrent chemoradiotherapy) (Supplementary Table 1). Treatment outcomes for cutaneous lymphomas and other rare subtypes of T-cell lymphomas were excluded from this analysis. Continuous variables were reported as medians with ranges, while categorical variables were summarized as frequencies and percentages. The chi-square test was used for categorical variables and the t-test was used for continuous variables in between-group comparisons. The relative survival rate (RSR) was estimated by comparing observed survival in patients with cancer to the expected survival in the general population [15, 16]. The 5-year RSR was calculated using the Ederer II method and the algorithm developed by Paul Dickman in SAS [17]. PFS was calculated from the date of diagnosis to the date of relapse, disease progression, death from any cause, or last follow-up. OS was measured from the date of diagnosis to death from any cause or last follow-up. PFS and OS probabilities were calculated using the Kaplan–Meier method and compared using the log-rank test. The Cox regression model was used to identify factors affecting long-term survival, including both patient-related and disease-related variables. Variables with a *p*-value < 0.1 in univariate analysis were included in the multivariate analysis using the stepwise selection method. For each factor, the hazard ratio (HR) and 95% confidence interval (CI) were calculated. A *p*-value < 0.05 was considered statistically significant. Statistical analyses were performed using SAS statistical software, version 9.4 (SAS Institute Inc., Cary, NC).

## Results

### Characteristics and relative survival of all patients

Out of 14,572 patients with T-cell lymphomas, 12,573 were included in this study. Data for 1,999 patients were either missing or unavailable. Supplementary Table 2 shows age and sex at diagnosis, as well as the distribution of different PTCL subtypes. Overall, the most common diagnoses occurred in patients aged 50–70 years, with the highest frequency in their 60s (Supplementary Figure 1A). In the PTCL-NOS and enteropathy-associated T-cell lymphoma (EATL) groups, the most frequent diagnoses were in patients in their 60s. In contrast, the AITL and ALK-negative ALCL groups had the highest frequency in patients in their 70s. EN NK/T-cell lymphoma was most frequently diagnosed in patients in their 50s (Supplementary Figure 1B). The proportion of males was higher than that of females, at 61% (Supplementary Figure 1C). In most subtypes, incidence was higher in male than in female patients (Supplementary Figure 1D). Among mature NK/T-cell lymphomas, PTCL-NOS (25.5%) and EN NK/T-cell lymphoma (24.9%) were the most frequently diagnosed, followed by AITL (16.7%). Among cutaneous-associated T-cell lymphomas, mycosis fungoides was the most common.

Supplementary Table 3 shows the 5-year RSR of EN NK/T-cell lymphoma and nodal T-cell lymphomas. Overall, RSR has improved over the past five years. Compared to the general population, the RSR for patients with ALK-positive ALCL was the highest, followed by those with EN NK/T-cell lymphoma. In contrast, the 5-year RSR for patients with EATL was low, ranging from 30.1% to 37.3%.

### Patients who underwent treatment for a cure

Table 1 shows the baseline characteristics of patients who received treatment. Of the 12,573 patients, 5,072 underwent chemotherapy or CCRT. After frontline chemotherapy, 741 (14.6%) patients underwent consolidative auto-SCT, 149 (2.9%) underwent allo-SCT, and 4,182 (82.5%) completed their initial treatment with chemotherapy or CRRT (Supplementary Figure 2). The median ages at diagnosis for patients who received auto-SCT and allo-SCT were 50 years (range, 40–58) and 45 years (range, 28–55), respectively, generally younger than those who did not undergo transplantation. In the non-transplant group, the highest proportion of patients were in their 60s and 70s, whereas in the transplant group, most were in their 40s and 50s. Overall, most patients were diagnosed with PTCL-NOS (33.7%) and EN NK/T-cell lymphoma (33.6%), followed by AITL (19.1%). Among patients who underwent auto- or allo-SCT, PTCL-NOS was the most common diagnosis. The CHOP-like regimen was most frequently used for nodal T-cell lymphomas. For extranodal lymphomas, the SMILE regimen was most commonly used, followed by CCRT (cisplatin plus radiotherapy) and VIPD (etoposide, ifosfamide, cisplatin, and dexamethasone).

**Table 1:** Characteristics of patients who underwent chemotherapy

Characteristics	Total No. (%)	None No. (%)	Auto-SCT No. (%)	Allo-SCT No. (%)	*p*-value
No. of patients	5,072 (100)	4,182 (82.5)	741 (14.6)	149 (2.9)	
Age, median (range), years	60 (48–70)	63 (50–72)	50 (40–58)	45 (28–55)	< 0.001
< 21	208 (4.1)	142 (3.4)	41 (5.5)	25 (16.8)	< 0.001
21–30	229 (4.5)	148 (3.5)	64 (8.6)	17 (11.4)	
31–40	362 (7.1)	247 (5.9)	97 (13.1)	18 (12.1)	
41–50	742 (14.6)	525 (12.6)	180 (24.3)	37 (24.8)	
51–60	1105 (21.8)	835 (20.0)	233 (31.4)	37 (24.8)	
61–70	1211 (23.9)	1070 (25.6)	126 (17.0)	15 (10.1)	
71–80	995 (19.6)	995 (23.8)	–	–	
> 80	220 (4.3)	220 (5.3)	–	–	
Sex					0.207
Male	3,167 (62.4)	2588 (61.9)	482 (65.0)	97 (65.1)	
Female	1,905 (37.6)	1594 (38.1)	259 (35.0)	52 (34.9)	
Diagnosis					< 0.001
PTCL, NOS	1,709 (33.7)	1,385 (33.1)	273 (36.8)	51 (34.2)	
EN NK/T-cell lymphoma	1,704 (33.6)	1,440 (34.5)	223 (30.1)	41 (27.5)	
AITL	967 (19.1)	786 (18.8)	157 (21.2)	24 (16.1)	
ALCL, ALK-positive	172 (3.4)	151 (3.6)	17 (2.3)	4 (2.7)	
ALCL, ALK-negative	114 (2.2)	95 (2.3)	16 (2.2)	3 (2.0)	
EATL	119 (2.3)	88 (2.1)	27 (3.6)	4 (2.7)	
Subcutaneous panniculitis-like T-cell lymphoma	57 (1.1)	45 (1.0)	7 (0.9)	5 (3.4)	
Primary cutaneous CD30-positive T-cell proliferation	56 (1.1)	50 (1.2)	4 (0.5)	2 (1.3)	
Mycosis fungoides	55 (1.1)	40 (1.2)	9 (1.2)	6 (4.0)	
Adult T-cell lymphoma/leukemia	51 (1.0)	45 (1.1)	3 (0.4)	3 (2.0)	
Prolymphocytic leukemia of T-cell Type	33 (0.7)	28 (0.6)	2 (0.2)	3 (2.0)	
Hepatosplenic T-cell lymphoma	22 (0.4)	16 (0.4)	3 (0.4)	3 (2.0)	
Sezary disease	13 (0.3)	13 (0.3)	–	–	
Frontline therapy					< 0.001
CVP	313 (6.2)	281 (6.7)	22 (3.0)	10 (6.7)	
CHOP	2859 (56.4)	2389 (57.1)	412 (55.6)	58 (38.9)	
CHOEP	577 (11.4)	431 (10.3)	115 (15.5)	31 (20.8)	
EPOCH	152 (3.0)	119 (2.8)	23 (3.1)	10 (6.7)	
VIPD	142 (2.8)	128 (3.1)	13 (1.8)	1 (0.7)	
SMILE	635 (12.5)	474 (11.3)	125 (16.9)	36 (24.2)	
Cisplatin + Radiotherapy	389 (7.7)	356 (8.5)	30 (4.0)	3 (2.0)	
Others	5 (0.1)	4 (0.1)	1 (0.1)	–	

*Abbreviations: allo-SCT* allogeneic stem cell transplantation, *auto-SCT* autologous stem cell transplantation, *PTCL* peripheral T-cell lymphoma, *NOS* not otherwise specified, *ALCL* anaplastic large cell lymphoma, *NK* natural killer, *AITL* angioimmunoblastic T-cell lymphoma, *EATL* enteropathy-associated T-cell lymphoma

### Long-term outcomes

**Fig. 1:**
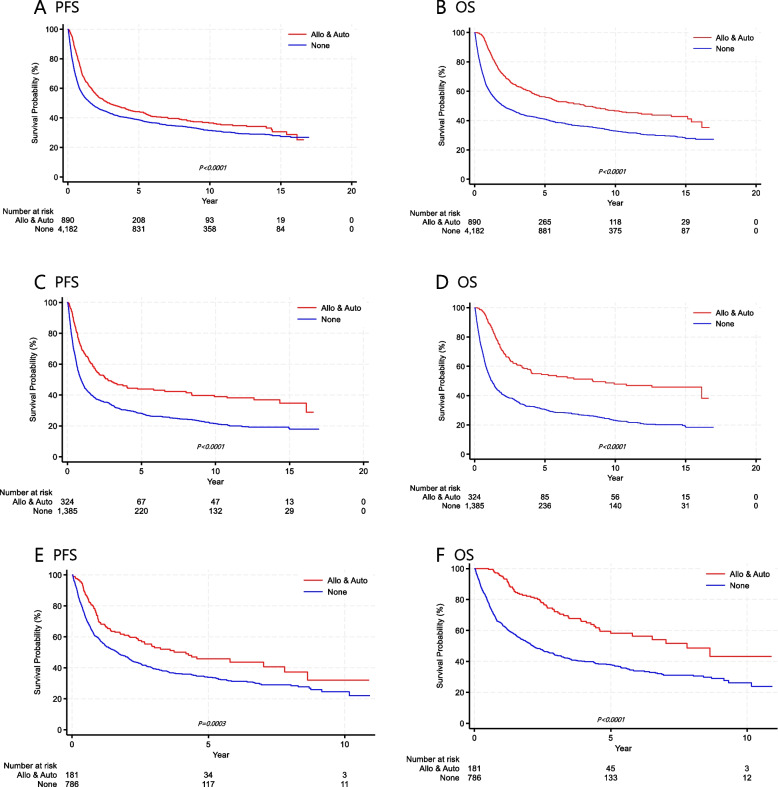
Kaplan–Meier survival curves. PFS (**A**) and OS (**B**) of all patients based on transplantation; PFS (**C**) and OS (**D**) in patients with PTCL-NOS based on transplantation; PFS (**E**) and OS (**F**) in patients with AITL based on transplantation

**Fig. 2:**
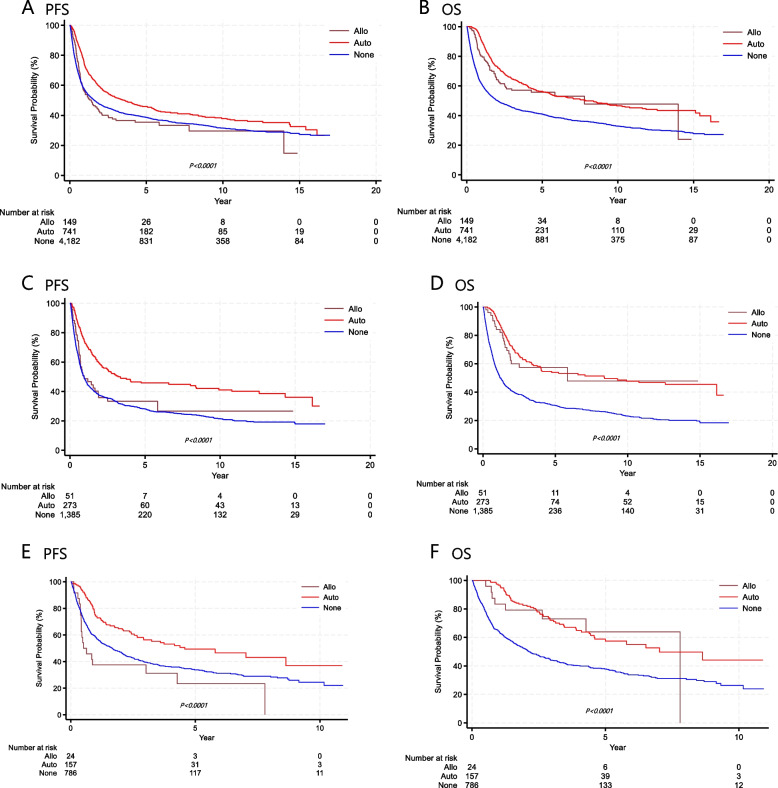
Kaplan–Meier survival curves. Comparison of PFS (**A**) and OS (**B**) between auto-SCT and allo-SCT in all patients; PFS (**C**) and OS (**D**) between auto-SCT and allo-SCT in patients with PTCL-NOS; PFS (**E**) and OS (**F**) between auto-SCT and allo-SCT in patients with AITL. Abbreviations: PFS, progression-free survival; OS, overall survival; AITL, angioimmunoblastic T-cell lymphoma

**Fig. 3:**
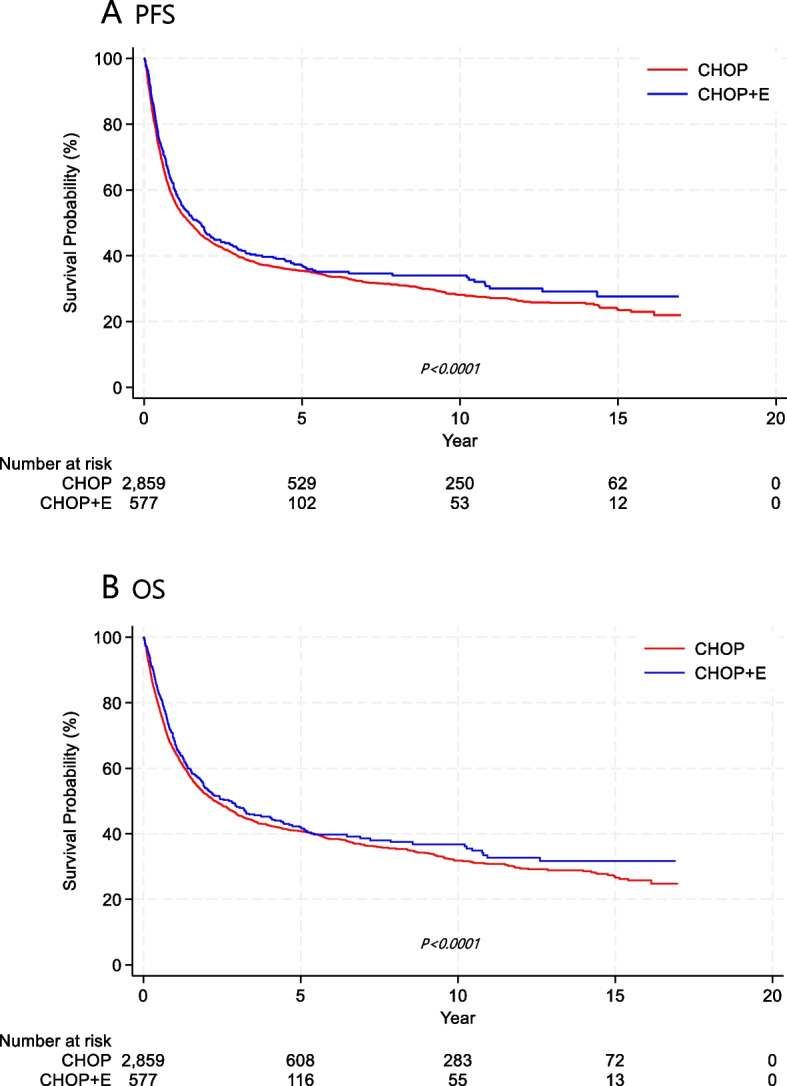
Kaplan–Meier survival curves. Patients who received etoposide in addition to the CHOP regimen showed statistically significantly improved PFS (**A**) and OS (**B**) compared to those treated with CHOP alone

The median follow-up duration was 6.7 years (range, 0.1–10.4). The 3-year and 5-year PFS rates for all treated patients were 44.0% and 39.5%, respectively, while the OS rates were 48.6% and 43.5%, respectively. Table 2 shows the PFS and OS of each subtype, comparing those who underwent auto-SCT or allo-SCT with those who did not receive transplantation. Kaplan–Meier survival curves indicated that patients who underwent transplantation had statistically significantly better PFS and OS than those who did not. The survival benefit of transplantation was especially evident in the PTCL-NOS and AITL subtypes (Fig. 1). In patients with EN NK/T-cell lymphoma and ALK-negative ALCL, transplantation also improved OS. However, allo-SCT did not show superiority over auto-SCT in consolidative up-front transplantation (Fig. 2). Among patients with nodal PTCLs, 2,859 received the CHOP regimen, while 577 received CHOP plus etoposide. Patients who added etoposide to the CHOP regimen showed statistically significantly improved PFS and OS in Kaplan–Meier survival curves (Fig. 3).

**Table 2:** PFS and OS of patients treated with curative intent

Subtype	Group	PFS		OS	
		3-year	5-year	3-year	5-year
All Patients	Total	44.0%	39.5%	48.6%	43.5%
	None	42.9%	38.5%	45.4%	40.8%
	Auto-SCT	51.7%	45.7%	60.1%	55.9%
	Allo-SCT	37.2%	35.0%	57.0%	55.6%
PTCL, NOS	Total	36.5%	31.1%	41.0%	35.1%
	None	33.5%	28.1%	36.1%	30.5%
	Auto-SCT	51.7%	45.5%	61.9%	53.8%
	Allo-SCT	32.9%	32.9%	57.5%	57.5%
EN NK/T-cell lymphoma	Total	51.3%	47.5%	53.9%	49.9%
	None	51.1%	47.7%	52.6%	49.0%
	Auto-SCT	54.7%	48.4%	65.1%	57.8%
	Allo-SCT	42.4%	42.4%	42.2%	42.2%
ALCL, ALK-positive	Total	84.9%	83.7%	88.8%	87.5%
	None	88.0%	86.6%	87.9%	86.5%
	Auto-SCT	63.3%	63.3%	93.9%	93.9%
	Allo-SCT	71.4%	71.4%	-	-
ALCL, ALK-negative	Total	35.6%	34.6%	45.7%	41.4%
	None	38.4%	36.3%	39.9%	39.9%
	Auto-SCT	47.9%	35.9%	80.7%	62.7%
	Allo-SCT	-	-	39.9%	-
AITL	Total	42.1%	36.0%	49.3%	41.6%
	None	39.3%	33.6%	43.8%	37.6%
	Auto-SCT	37.5%	23.0%	71.6%	57.1%
	Allo-SCT	56.0%	48.9%	73.2%	63.4%

*Abbreviations: allo-SCT* allogeneic stem cell transplantation, *auto-SCT* autologous stem cell transplantation, *PFS* progression-free survival, *OS* overall survival, *AITL* angioimmunoblastic T-cell lymphoma, *PTCL* peripheral T-cell lymphoma, *NOS* not otherwise specified, *ALCL* anaplastic large cell lymphoma, *NK* natural killer

## Discussion

We evaluated the epidemiology and long-term survival outcomes of Korean patients with PTCLs using NHIS data from the past 15 years. In this study, the incidence of mature T-cell and NK-cell lymphomas was higher in males and generally more common in individuals in their 60s. PTCL-NOS and EN NK/T-cell lymphoma were the most frequently diagnosed subtypes. Patients with ALK-positive ALCL showed superior long-term survival compared to other investigated subtypes. Treatment outcomes were better in patients who received etoposide, including CHOP-based regimens. The survival benefits of consolidative transplantation were confirmed in PTCL-NOS and AITL.

According to the WHO classification, PTCLs are categorized into nodal, extranodal, leukemic, and primary cutaneous T-cell lymphomas [3, 4]. The incidence of PTCL subtypes varies geographically. In the International T-cell Lymphoma Project, PTCL-NOS was the predominant subtype in North America and Europe, whereas EN NK/T-cell lymphoma and adult T-cell leukemia/lymphoma were more prevalent in Asia [18]. In the Asian NK/T-cell lymphoma registry, the incidence of EN NK/T-cell lymphoma was highest at 28.6%, followed by AITL (24.7%) and PTCL-NOS (20.8%) [19]. In our study, PTCL-NOS was the most common subtype, followed by EN NK/T-cell lymphoma and AITL.

The rarity and heterogeneity of PTCLs limit clinical studies on treatment. Several attempts have been made regarding frontline therapy for nodal PTCLs since the landmark SWOG phase III study established CHOP as the standard chemotherapy [20]. Adding etoposide to CHOP (CHOEP) was initially explored by the German High-Grade Non-Hodgkin Lymphoma Study Group, where CHOEP improved event-free survival in young, low-risk patients. Some studies showed survival advantages of CHOEP over CHOP in PFS and/or OS, particularly in patients with ALK-positive ALCL. However, CHOEP has primarily been used in patients under 60–65 years of age due to concerns about toxicity [21–24]. Inconsistent results have also been reported. In a multivariate analysis using the Netherlands Cancer Registry, the absence of up-front auto-SCT was associated with inferior OS, while the use of CHOEP had no significant impact on survival [19]. While the evidence for CHOEP is strongest in ALK-positive ALCL, BV plus CHP (cyclophosphamide, doxorubicin, and prednisolone) has largely replaced its use based on the ECHELO-2 study [9]. Combination regimens of CHOP with novel agents such as alemtuzumab and romidepsin, as well as intensive regimens such as DA-EPOCH (dose-adjusted etoposide, prednisone, vincristine, cyclophosphamide, and doxorubicin), Mega-CHOEP, and Hyper-CVAD (cyclophosphamide, vincristine, doxorubicin, dexamethasone/methotrexate, and cytarabine), have been investigated in untreated PTCLs. However, these regimens did not demonstrate survival benefits over CHOP/CHOEP and often resulted in greater toxicity [25–28].

The lack of randomized controlled trials on transplantation in first remission, coupled with PTCL heterogeneity, makes it challenging to establish a clear role for consolidative up-front transplantation. Several studies have reported survival benefits of up-front auto-SCT following frontline therapy [5, 29–31]. The largest prospective study, conducted by the Nordic Lymphoma Group (NGL-T-01), enrolled 115 patients with nodal PTCL (excluding ALK-positive ALCL), of whom 70% underwent auto-SCT. The 5-year PFS and OS rates were 44% and 51%, respectively—better than historical controls [29]. Outcomes for historically poor-prognosis subtypes like AITL and PTCL-NOS have improved with more consistent use of auto-SCT [32, 33]. However, some studies have not confirmed the benefit of up-front auto-SCT [8, 23, 34, 35]. A retrospective study by the LYSA group showed no PFS or OS benefit of auto-SCT in multivariate analysis or with propensity score matching [34]. The LYSA and GLA study groups evaluated the outcomes of allo-SCT and auto-SCT as up-front consolidative treatments in newly diagnosed PTCLs. While allo-SCT offered an advantage in relapse settings, it conferred no survival benefit due to high transplant-related mortality [13]. One study showed that patients who underwent auto-SCT in complete or partial remission had significantly better OS than those who received allo-SCT [14].

Although this study included a relatively large patient cohort and yielded meaningful results, it has some limitations requiring cautious interpretation. First, patients were classified using KCD codes from NHIS, and due to the volume of data, histopathological confirmation could not be performed—introducing a potential misclassification bias. Second, the retrospective nature of the cohort carries inherent limitations. Detailed baseline data such as disease stage, severity, and performance status—which substantially affect prognosis—were not available for analysis. Third, the criteria used to determine eligibility for transplantation, as well as the selection between auto- and allo-SCT, were not specified.

In summary, we investigated the epidemiology of mature T- and NK-cell lymphomas and long-term survival outcomes by subtype. Patients with ALK-positive ALCL had relatively superior outcomes. Long-term benefits were observed when CHOP plus etoposide was used as first-line therapy. Up-front transplantation showed survival advantages for PTCL-NOS and AITL, with no evidence supporting the superiority of allo-SCT over auto-SCT. Decisions regarding frontline therapy and transplantation should be individualized by subtype. Larger prospective randomized studies are needed to establish unified treatment guidelines.

## Supplementary Information

Supplementary Material 1.

## Data Availability

The datasets generated and/or analyzed during the current study are available from the corresponding author on reasonable request.
