# Supplementary material for: Real-world data on long-term outcomes in patients with T-cell lymphomas: a nationwide study of Korea
Source: Blood Res. 2025 Aug 21;60(1):47. doi: 10.1007/s44313-025-00095-1 (PMC12370571; doi:10.1007/s44313-025-00095-1)
Supplement: Supplementary file 1 — Supplementary Material 1. [file 44313_2025_95_MOESM1_ESM.docx]

**Supplementary materials**

**Supplementary Table 1.** Components of each chemotherapy

| Regimens | Components |
| --- | --- |
| CVP | cyclophosphamide, vincristine, and prednisone |
| CHOP | cyclophosphamide, doxorubicin or adriamycin, vincristine, and prednisone |
| CHOEP | cyclophosphamide, doxorubicin or adriamycin, vincristine, etoposide, and prednisone |
| EPOCH | etoposide, prednisone, vincristine, cyclophosphamide, and doxorubicin or adriamycin |
| VIPD | etoposide, ifosfamide, cisplatin, and dexamethasone |
| SMILE | dexamethasone, methotrexate, ifosfamide, L-asparaginase, and etoposide |
| CCRT | concurrent chemoradiothearpy (cisplatin plus radiotherapy) |

**Supplementary Table 2**. Baseline characteristics of patients (n = 12,573)

| Characteristics | No. (%) |
| --- | --- |
| Age at the time of diagnosis, years |  |
| < 10 | 152 (1.2) |
| 10–19 | 495 (3.9) |
| 20–29 | 781 (6.2) |
| 30–39 | 1,096 (8.7) |
| 40–49 | 1,767 (14.1) |
| 50–59 | 2,452 (19.5) |
| 60–69 | 2,598 (20.7) |
| 70–79 | 2,310 (18.4) |
| > 80 | 922 (7.3) |
| Sex |  |
| Male | 4,958 (39.4) |
| Female | 7,615 (60.6) |
| Histopathology |  |
| PTCL, NOS | 3200 (25.5) |
| Extranodal NK/T-cell lymphoma, nasal type | 3130 (24.9) |
| AITL | 2096 (16.7) |
| ALCL, ALK (+) | 396 (3.1) |
| ALCL, ALK (-) | 328 (2.6) |
| EATL | 207 (1.6) |
| Hepatosplenic T-cell lymphoma | 63 (0.5) |
| Mature T/NK-cell lymphoma, unspecified | 779 (6.2) |
| Mycosis fungoides | 624 (5.0) |
| Subcutaneous panniculitis-like T-cell lymphoma | 115 (0.9) |
| Primary cutaneous CD30-positive T-cell proliferation | 107 (0.9) |
| Sezary disease | 29 (0.2) |
| Cutaneous T-cell lymphoma, unspecified | 1152 (9.2) |
| Adult T-cell lymphoma/leukemia | 240 (1.9) |
| T-cell prolymphocytic leukemia | 107 (0.9) |

Abbreviations: PTCL, Peripheral T-cell lymphoma; NOS, not otherwise specified; ALCL, anaplastic large cell lymphoma; NK, natural killer; AITL, angioimmunoblastic T-cell lymphoma; EATL, Enteropathy-associated T-cell lymphoma

**Supplementary Table 3**. Five-year relative survival rates by age group

| Subtype | Age (yr) | 2011–2015 | 2016–2020 |
| --- | --- | --- | --- |
| Extranodal NK/T-cell lymphoma, nasal type | Total | 70.3 | 72.5 |
|  | 0–14 | - | - |
|  | 15–34 | 71.6 | 77.5 |
|  | 35–49 | 78.4 | 80.6 |
|  | 50–64 | 74.1 | 74.4 |
|  | 65–79 | 57.9 | 71.3 |
|  | 80+ | 72.6 | 53.6 |
| ALCL, ALK-positive | Total | 86.8 | 93.5 |
|  | 0–14 | - | 93.8 |
|  | 15–34 | 94.2 | 96.0 |
|  | 35–49 | 86.8 | 97.2 |
|  | 50–64 | 80.0 | 93.7 |
|  | 65–79 | 80.6 | 83.2 |
|  | 80+ | - | 61.8 |
| ALCL, ALK-negative | Total | 60.7 | 77.5 |
|  | 0–14 | - | 90.1 |
|  | 15–34 | 86.0 | 97.2 |
|  | 35–49 | 64.2 | 80.2 |
|  | 50–64 | 81.1 | 72.3 |
|  | 65–79 | 39.3 | 65.8 |
|  | 80+ | 56.4 | - |
| AITL | Total | 54.7 | 63.8 |
|  | 0–14 | - | - |
|  | 15–34 | 88.5 | 72.9 |
|  | 35–49 | 72.5 | 86.2 |
|  | 50–64 | 64.7 | 70.1 |
|  | 65–79 | 48.1 | 61.0 |
|  | 80+ | 39.6 | 45.3 |
| Hepatosplenic T-cell lymphoma | Total | 57.9 | 49.1 |
|  | 0–14 | - | - |
|  | 15–34 | 75.1 | 50.2 |
|  | 35–49 | 40.3 | 71.9 |
|  | 50–64 | 76.7 | 45.4 |
|  | 65-79 | 56.7 | 21.6 |
|  | 80+ | - | - |
| EATL | Total | 30.1 | 37.3 |
|  | 0–14 | - | - |
|  | 15–34 | - | - |
|  | 35–49 | 27.5 | 46.0 |
|  | 50–64 | 24.5 | 37.2 |
|  | 65–79 | 43.5 | 25.2 |
|  | 80+ | 18.1 | - |
| PTCL, NOS | Total | 54.4 | 61.1 |
|  | 0–14 | 87.6 | 85.1 |
|  | 15–34 | 73.0 | 81.3 |
|  | 35–49 | 78.8 | 72.8 |
|  | 50–64 | 55.7 | 61.5 |
|  | 65–79 | 47.3 | 52.4 |
|  | 80+ | 40.7 | 47.2 |

Abbreviations: allo-SCT, allogeneic stem cell transplantation; Auto-SCT, autologous stem cell transplantation; PTCL, Peripheral T-cell lymphoma; NOS, not otherwise specified; ALCL, anaplastic large cell lymphoma; NK, natural killer; AITL, angioimmunoblastic T-cell lymphoma; EATL, Enteropathy-associated T-cell lymphoma

**Supplementary Figure 1**. Epidemiology of mature T-cell and NK-cell lymphomas

1. Incidence of PTCLs according to age


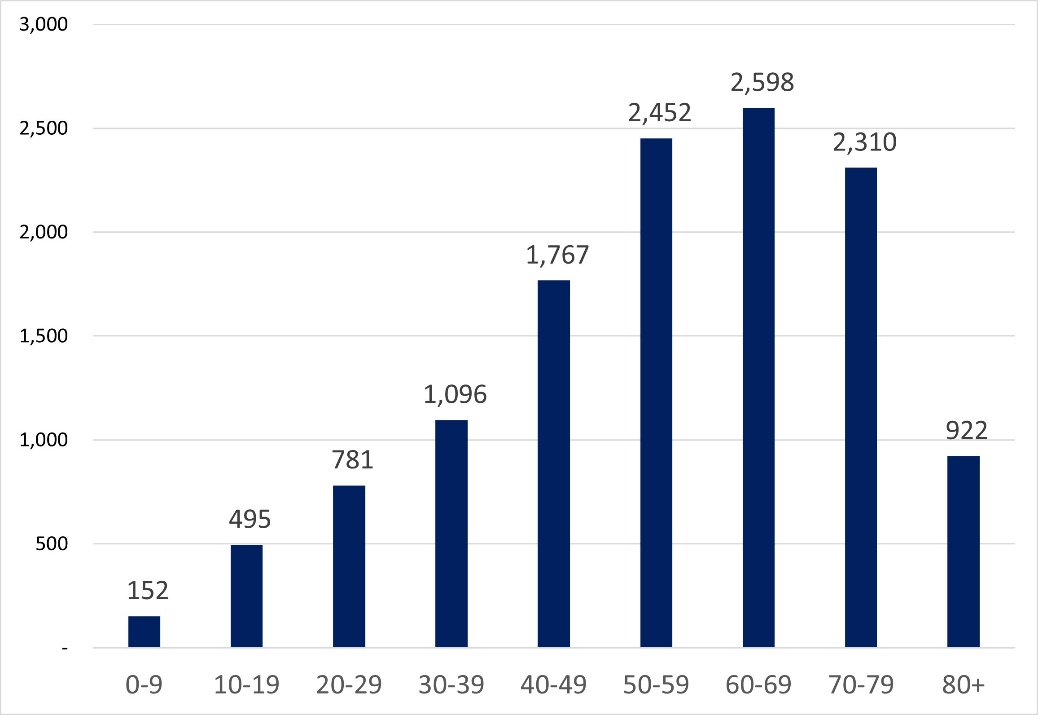


(B) Age distribution at diagnosis of each subtype of PTCLs


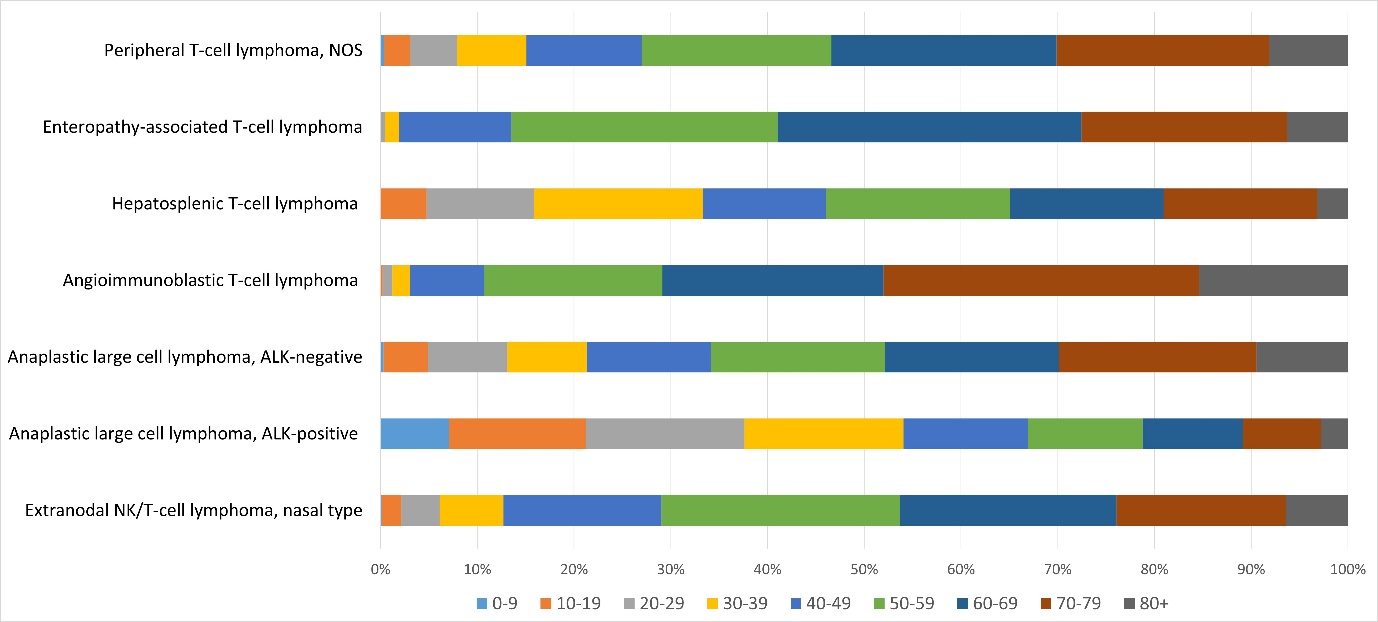


(C) Sex ratio of PTCLs


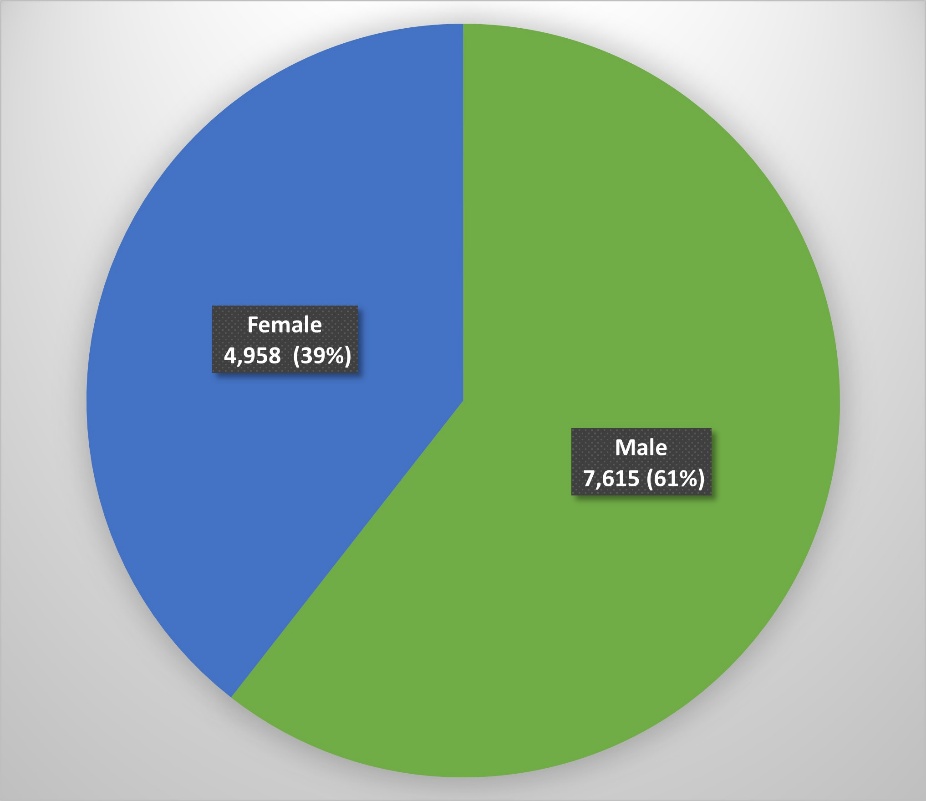


(D) Sex ratio according to each subtype of PTCLs


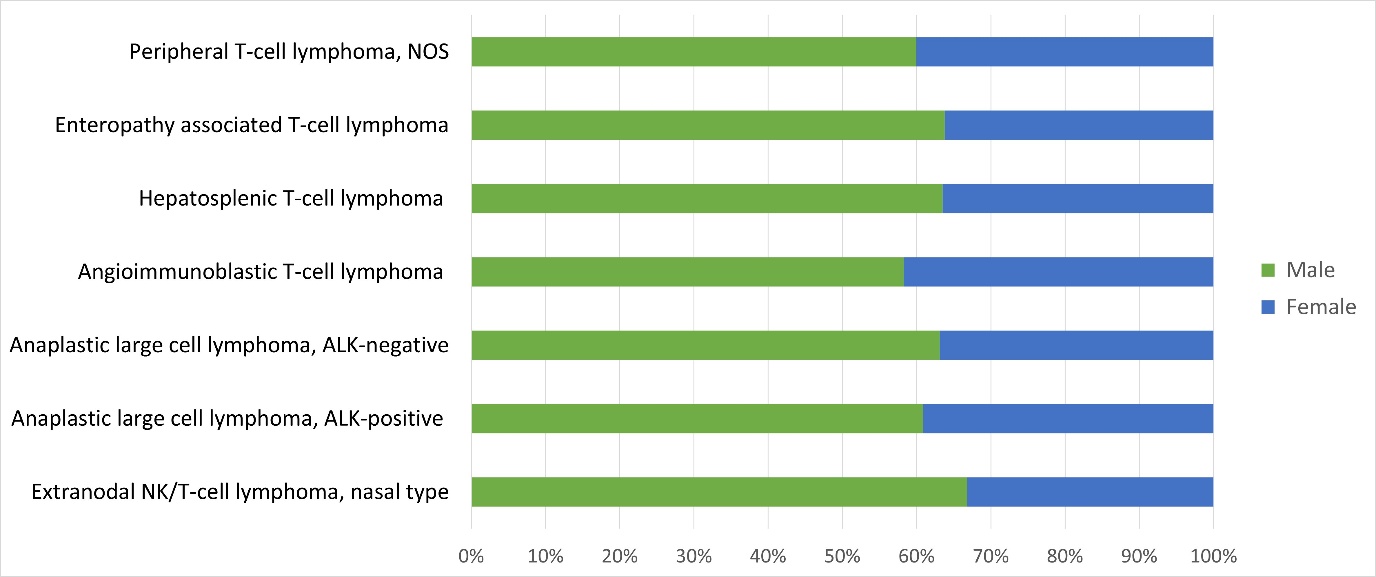


Abbreviations: PTCLs, Peripheral T-cell lymphomas; NOS, not otherwise specified; ALCL, anaplastic large cell lymphoma; NK, natural killer; AITL, angioimmunoblastic T-cell lymphoma; EATL, Enteropathy-associated T-cell lymphoma

**Supplementary Figure 2**. Consort diagram of the patients who received treatment


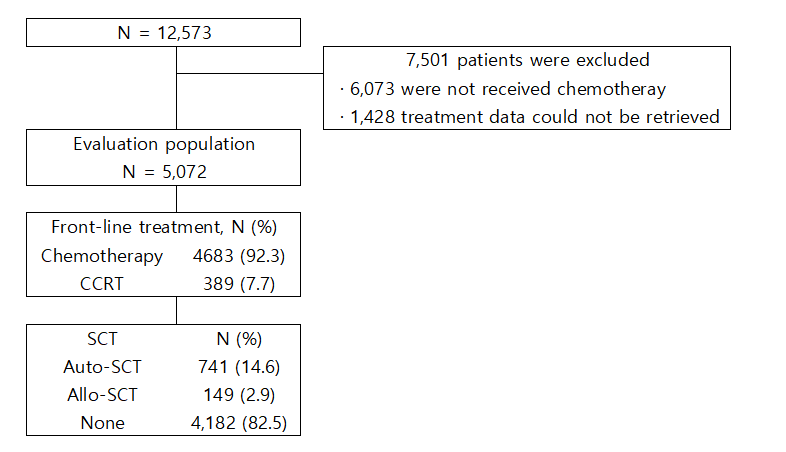


Abbreviations: allo-SCT, allogeneic stem cell transplantation; CCRT, concurrent chemo-radiotherapy; SCT, stem cell transplantation
